# The double fascicular variations of the anterior talofibular ligament and the calcaneofibular ligament correlate with interconnections between lateral ankle structures revealed on magnetic resonance imaging

**DOI:** 10.1038/s41598-020-77856-8

**Published:** 2020-11-27

**Authors:** Paweł Szaro, Khaldun Ghali Gataa, Mateusz Polaczek, Bogdan Ciszek

**Affiliations:** 1 Department of Radiology, Institute of Clinical Sciences, Sahlgrenska Academy, University of Gothenburg, Gothenburg, Sweden; 2 Department of Musculoskeletal Radiology, Sahlgrenska University Hospital, Göteborgsvägen 31, 431 80 Gothenburg, Sweden; 3 Medical University of Warsaw, Warsaw, Poland; 4 Department of Descriptive and Clinical Anatomy, Medical University of Warsaw, Warsaw, Poland

**Keywords:** Ligaments, Muscle, Skeleton

## Abstract

The anterior talofibular ligament and the calcaneofibular ligament are the most commonly injured ankle ligaments. This study aimed to investigate if the double fascicular anterior talofibular ligament and the calcaneofibular ligament are associated with the presence of interconnections between those two ligaments and connections with non-ligamentous structures. A retrospective re-evaluation of 198 magnetic resonance imaging examinations of the ankle joint was conducted. The correlation between the double fascicular anterior talofibular ligament and calcaneofibular ligament and connections with the superior peroneal retinaculum, the peroneal tendon sheath, the tibiofibular ligaments, and the inferior extensor retinaculum was studied. The relationships between the anterior talofibular ligament’s and the calcaneofibular ligament’s diameters with the presence of connections were investigated. Most of the connections were visible in a group of double fascicular ligaments. Most often, one was between the anterior talofibular ligament and calcaneofibular ligament (74.7%). Statistically significant differences between groups of single and double fascicular ligaments were visible in groups of connections between the anterior talofibular ligament and the peroneal tendon sheath (*p* < 0.001) as well as the calcaneofibular ligament and the posterior tibiofibular ligament (*p* < 0.05), superior peroneal retinaculum (*p* < 0.001), and peroneal tendon sheath (*p* < 0.001). Differences between the thickness of the anterior talofibular ligament and the calcaneofibular ligament (*p* < 0.001), the diameter of the fibular insertion of the anterior talofibular ligament (*p* < 0.001), the diameter of calcaneal attachment of the calcaneofibular ligament (*p* < 0.05), and tibiocalcaneal angle (*p* < 0.01) were statistically significant. The presence of the double fascicular anterior talofibular ligament and the calcaneofibular ligament fascicles correlate with connections to adjacent structures.

## Introduction

The ATFL is the weakest part of the lateral ankle ligaments, which is usually the first and most common injured ligament in ankle inversions. The anterior talofibular ligament (ATFL) is composed of the superior (S-ATFL) and inferior fascicle (I-ATFL)^1–7^. Meanwhile, the calcaneofibular ligament (CFL) is composed of the medial (M-CFL) and lateral (L-CFL)^6,8,9^. The interconnections between the ATFL, CFL, and the posteriori talofibular ligament (PTFL) provide integration between components making a complex of ligaments to a functional unit^1,10^. The most prominent interconnection is I-ATFL and the CFL, which is usually arch-formed^11^. The S-ATFL is considered as an intraarticular structure, while the I-ATFL is an extraarticular part. It is unclear if the S-ATFL is intrasynovial or only intracapsular. If the only S-ATFL is injured, healing potential is lower because of its intraarticular localization, and if the I-ATFL is preserved, a secondary microinstability may appear^12^. However, during injury propagating via the I-ATFL, forces may also be transmitted to the CFL, causing its injury, and the full instability may appear^13^.

The diagnostic imaging of the posttraumatic ankle is based mainly on the clinical examination and x-ray. However, MRI is often performed to accurately assess all injuries to bones and soft tissue^14^. MRI criteria of injury to a ligament also include an assessment of dimensions; however, the cut-off values are not fully established. There is a significant discrepancy in the length, thickness, and width of the normal ATFL (Table 1) and CFL (Table 2). Most of the previously published anatomical research based on dissections focuses on surgically useful measurements as length and width. However, studies on thickness measurement are found less frequently. The mean thickness of the ATFL measured on MRI was 2.19 ± 0.6 mm, and the corresponding value for the CFL was 2.13 ± 0.5 mm^15^. According to an MRI study, the length of the ATFL was 2.19 mm, and the respective value for CFL was 2.13 mm^16^. The definition of the normal diameters is essential in the assessment of the ATFL and CFL after trauma and in chronic instability^17^.

**Table 1:** Comparing the measurements of length, width, and thickness of the ATFL.

study	N	length (mm)	width (mm)	thickness (mm)	fibular insertion CC (mm)	talar insertion CC (mm)
Neuschwander et al.^18^	8	19.7 ± 1.2 (1)	–	–	–	–
		16.7 ± 1.1 (2)				
Clanton et al.^21^	14	16.3 (3)	–	–	–	–
		16.4 (1)				
		14.7 (2)				
Wenny et al.^9^	17	12.85 ± 2.64 (1)	6.62 ± 1.39			
		11.38 ± 2.25 (2)				
Taser et al.^22^	42	22.37 ± 2.50	10.77 ± 1.56 (4)	–	–	–
			6.75 ± 2.89 (5)			
			10.96 ± 2.38 (6)			
Siegler et al.^4^	20	17.81 ± 3.05	–	–	–	–
Milner and Soames^2^	40	13.0 ± 3.9	11.0 ± 3.3	–	–	–
Sindel et al.^23^	24	19.1 ± 2.28 (1)	6.7 ± 1.06 (1)	–	7.5 ± 1.32	6 ± 0.99
		15.2 ± 2.62 (2)	4.5 ± 1.09 (2)			
Uğurlu et al.^5^	22	20.84 (3)	7.61 (3)	–	–	–
		18.74 (1)	4.92 (1)			
		15.33 (2)	5.39 (2)			
Raheem, O’Brien^3^	20	15.5	–	–	–	–
Yıldız and Yalcın^7^	46	14.19 ± 2.02(1)	11.07 ± 5.63	–	–	–
		12.24 ± 1.99(2)				
Edama et al.^1^	81	21.3 ± 2.8 (3)	7.5 ± 2.6 (4)	–	–	–
		21.0 ± 2.3 (1)	6.1 ± 1.8 (5)			
		18.3 ± 2.8 (2)	7.1 ± 1.8 (6)			

The one value corresponds to the average diameter. When more than one values: (1) the S-ATFL, (2) the I-ATFL, (3) single fascicular ligament, (4) proximal, (5) middle, and (6) distal.

**Table 2:** Comparing the measurements of length, width, and thickness of the CFL.

Study	N	Length (mm)	Width (mm)	Thickness (mm)	Fibular insertion CC (mm)	Talar insertion CC (mm)
Kobayashi et al.^8^	181	23.9 ± 3.5 (1)	8.4 ± 2.3 (1)			
		19.8 ± 3.5 (2)	6.1 ± 1.5 (2)			
Clanton et. al.^21^	14	24.7	–	–	–	–
Neuschwander et al.^18^	8	24.8 ± 2.4	–	–	–	–
Raheem, O’Brien^3^	20	18.5	–	–	–	–
Siegler et al.^4^	20	27.69 ± 3.30	–	–	–	–
Sindel et al.^23^	24	26.8 ± 4.91	6 ± 0.8	–	–	–
Uğurlu et al.^5^	22	26.67	4.57	–	–	–
Wenny et al.^9^	17	20.88 ± 2.72 (4)	7.66 ± 1.68	–	–	–
		21.59 ± 2.70 (5)				
Yıldız and Yalcın^7^	46	15.03 ± 2.93	5.44 ± 2.34	–	–	–
		20.02 ± 2.99				

The one value corresponds to the average diameter. When more than one values: (1) single fascicular CFL, (2) two fascicular CFL, (3j) average, (4c) the superior outline, and (5d) the inferior outline.

There are also biomechanical differences between fascicles of the ATFL; the S-ATFL is taut in plantarflexion while the I-ATFL in dorsiflexion. Taking into consideration the biomechanical features of the ATFL, a possible two fascicle reconstruction may restore both the structure and function of the ligament. It should be added that such reconstruction is not performed nowadays^18^. The lateral ligament complex may include an occurring variable, the lateral talocalcaneal ligament, which may support the CFL. Malalignment of the ankle or subtalar joint may cause overuse of the supporting ligaments. Hindfoot valgus may influence the CFL^19^, which via existing interconnections, may cause dysfunction of the whole lateral ligament complex. Clinical observation indicates that the ATFL and CFL accompany the pathology of adjacent non-ligamentous structures as the superior peroneal retinaculum or the peroneal tendons. Morphological studies of anatomical connections may help in improving diagnostic imaging and surgery of the injured ligaments and non-ligamentous structures by understanding the mechanism of injury and by selecting the best method of reconstruction^20^. The hypothesis of our study is that there are interconnections between the lateral ligaments and non-ligamentous structures in the area of the lateral malleolus. The aim of the study was to assess the presence of communications between the ATFL and CFL with adjacent structures and correlate it with the ATFL and CFL’s morphology.

## Material and methods

The MRI examinations were done from January 2017 to April 2019, and no traumatic indications were reviewed. Exclusion criteria were trauma in the anamnesis, obvious abnormality in the lateral region of the ankle, the presence of the orthopedic hardware due to the possible artifacts (23 cases excluded), a history of previous fracture (19 cases excluded), and obvious abnormality in relation to the lateral malleolus (8 cases excluded). In total, 198 MRI examinations of the ankle fulfilled the inclusion and exclusion criteria. All examinations were reviewed twice, and the final results were made by consensus.

The MRI protocol may vary between cases, but inclusion criteria were the presence at least proton density (PD) or T2-weighted sequences without fat saturation in sagittal, axial, and coronal plane to assess ligaments structure. The other sequences, like T1-weighted turbo spin-echo (T1-TSE), PD with fat suppression, or short-T1 inversion recovery (STIR), were used to detect pathology, which might be the exclusion criterion. All MRI examinations were performed using the MRI machine Ingenia 3.0 T MR system (Philips Healthcare) with a dedicated ankle coil.

Our study included 110 females and 80 males with an age range of 18–58 years, and a mean age of 31 years. The right ankle was examined in 107 cases, and the left one in 91.

The statistical correlations between of presence of connection to adjacent structures, the morphometry, side, gender, and presence of the interconnections between the ATFL and CFL in the groups of single and double fascicular ATFL and CFL were analyzed. The other lateral structures included in our study were the PTFL, the superior peroneal retinaculum (SPR), the peroneal tendon sheath (PTS), the fibulotalocalcaneal ligament (FTCL), the anterior inferior tibio-fibular ligament (AITFL), and the inferior extensor retinaculum (IER). The measurement of the tibiocalcaneal angle (TCA), according to Buck et al.^24^ was performed and correlated with CFL morphology and morphometry.

**Figure 1:**
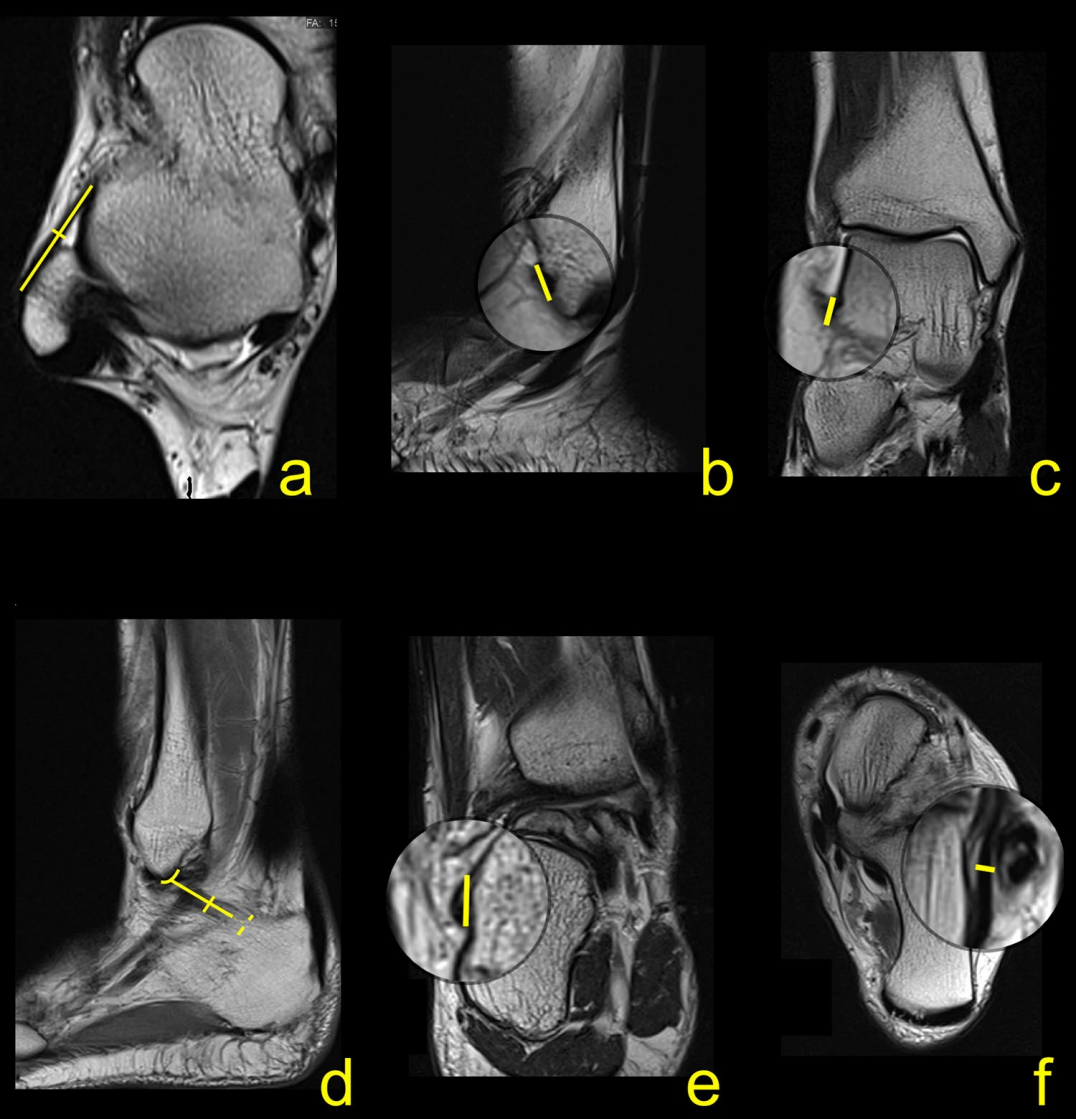
The protocol of the measurements a-f. Explanation in the section Material and Methods.

The protocol and some of the measurements are presented in Fig. 1. The length and thickness of the midportion of the ATFL was measured on the axial sequence (Fig. 1a). The length of the fibular insertion of the ATFL was evaluated on the sagittal sequence (Fig. 1b), while the coronal sequence was used to assess the biggest diameter of the talar insertion (Fig. 1c). The length, the width, and the diameter of the fibular insertion of the CFL were assessed on the consecutive sagittal slides (Fig. 1d). The coronal sequences were used to measure the length of the calcaneal insertion (Fig. 1e), while the thickness of the midportion was measured on the axial sequence in the middle part of the ligament (Fig. 1f). In the two-fascicular ligaments, the width was measured for both bundles together, and the ratio size between the fascicles was noted. The CFL angle and the TCA were measured as in a previous study^24^.

The Local Swedish Ethics Committee and Institutional Ethics Committee at the Medical University of Warsaw approved the study and waived the need for informed consent (Numbers 06177 and AKBE/258/2019) due to the retrospective and non-invasive nature of the study. The study was conducted in accordance with the Declaration of Helsinki. The anonymization of patient data in the research process ensured data protection in accordance with the European General Data Protection Regulation. The authors declare that this work has not received any funding before or during research. There are no relationships with any companies whose products or services may be related to the subject matter of the article.

## Results

**Figure 2:**
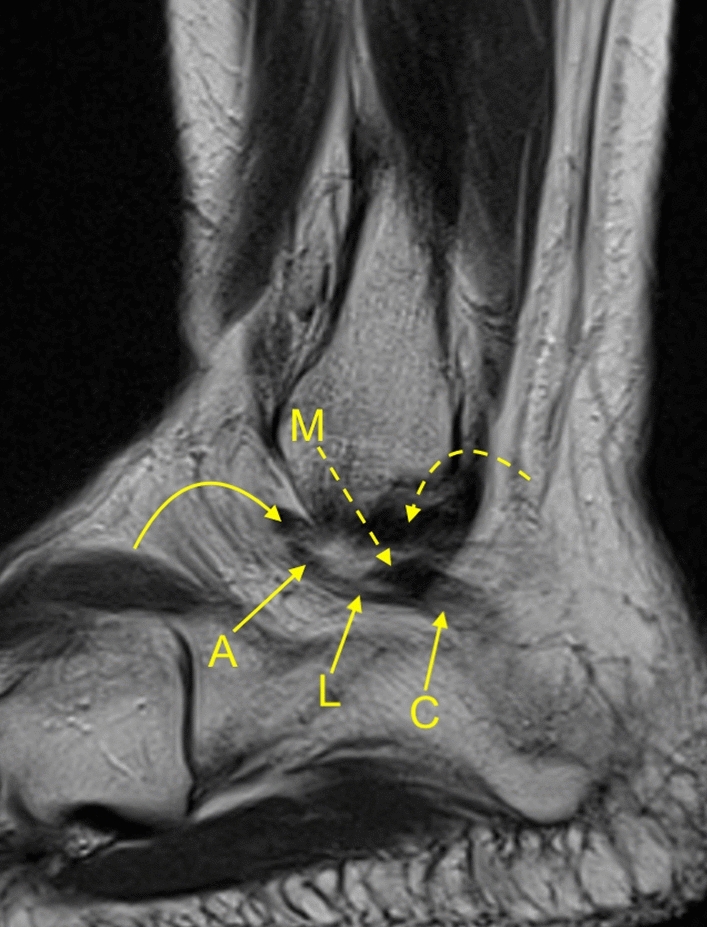
A 37-yeard-old patient with suspicion of a stress fracture of the navicular bone, which was not confirmed on the MRI. The arch-shaped communication between the ATFL (A), the L-CFL (L), the M-CFL (M), and the PTFL (curved dashed arrow) on the sagittal section.

**Figure 3:**
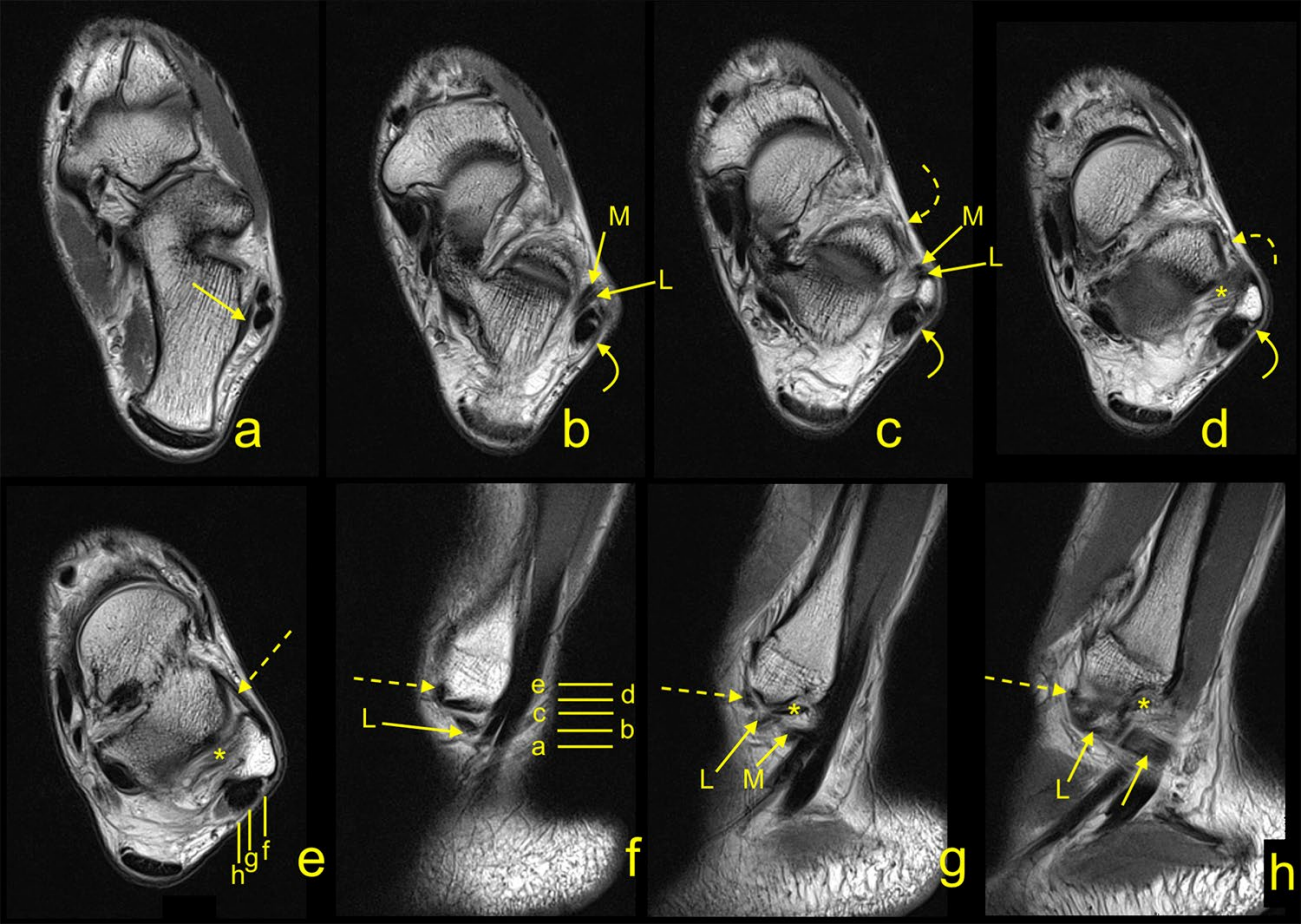
A 33-yeard-old patient with suspicion of an osteochondral lesion of the talus, which was not confirmed on the MRI. The communications present in relation to the lateral malleolus. The CFL (straight arrow), the ATFL (dashed arrow), the SPR (curved arrow), the IER (curved dashed arrow), and the PTFL (*). On figure (**b**, **c**, **f**, **g** and **h**) two fascicles of the CFL are visible: L- the L-CFL and M- the M-CFL. Levels of cross-sections are labeled on figures (**e**) and (**f**).

Our MRI study revealed the presence of interconnections between the ATFL and CFL and with other lateral ankle structures included in the study (Figs. 2 and 3). We noticed several statistically significant differences in the incidence of these connections and diameters between the groups of single and double fascicular ATFL and CFL (Tables 3 and 4). There were no significant differences regarding gender and side (*p* > 0.05).

**Table 3:** The number of direct connections between the structures included in the study in groups of single and double fascicular ligaments.

Structures:		CFL	PTFL	SPR	PTS	FTCL	AITFL	IER
**ATFL**								
Single fascicular	n = 49	23	5	5	0	9	4	11
	%	11.6%	2.5%	2.5%	0.0%	4.5%	2.0%	5.6%
Double fascicular	n = 148	125	11	53	29	12	9	48
	%	63.1%	5.6%	26.8%	14.6%	6.1%	4.5%	24.2%
Total	N = 198	148	16	58	29	21	13	59
	%	74.7%	8.1%	29.3%	14.6%	10.6%	6.6%	29.8%
*P* value		*p* > .05	*p* > .05	*p* > .05	*p* < .001	*p* > .05	*p* > .05	*p* > .05

Structures		ATFL	PTFL	SPR	PTS	FTCL	AITFL	IER
**CFL**								
Single fascicular	n = 67	43	31	47	42	18	0	0
		21.7%	15.7%	23.7%	21.2%	9.1%	0.0%	0.0%
Double fascicular	n = 131	105	105	86	2	109	0	0
	%	53.0%	53.0%	43.4%	1.0%	55.1%	0.0%	0.0%
Total	N = 198	148	136	133	44	127	0	0
	%	74.7%	68.7%	67.2%	22.2%	64.1%	0.0%	0,0%
*P* value		*p* > .05	*p* < .05	*p* < .001	*p* < .001	*p* > .05		

The abbreviations used in Table 1. AITFL, the anterior inferior tibio-fibular ligament; ATFL, the anterior talofibular ligament; CFL, the calcaneofibular ligament; FTCL, the fibulotalocalcaneal ligament; IER, the inferior extensor retinaculum; LTCL, the lateral talocalcaneal ligament; PTFL, the posteriori talofibular ligament; PTS, the peroneal tendon sheath; SPR, the superior peroneal retinaculum.

**Table 4:** The morphometry of the ATFL and its bone attachments in the total number of ATFL and groups of single and double fascicular ATFL.

Groups	Features	Length (mm)	Thickness of the midportion (mm)	The craniocaudal diameter of the midportion (mm)	Length of the fibular insertion (mm)	Length of the talar insertion (mm)
Totally ATFL	Average diameter	24.2	2.1	5.1	5.8	4.3
	Minimum	16.1	1.3	3.4	3.7	3.1
	Maximum	32.8	2.9	6.9	7.2	5.6
	SD	3.4	0.3	0.7	0.9	0.6
Single fascicular ATFL	Average	23.4	2.4	5.1	6.2	4.3
	Min	16.1	1.9	4.3	4.8	3.5
	Max	27.9	2.9	6.9	6.9	5.2
	SD	3.6	0.4	0.8	0.7	0.6
Double fascicular ATFL	Average	24.5	2.1	5.0	5.6	4.3
	Min	17.2	1.3	3.4	3.7	3.1
	Max	32.8	2.8	6.7	7.2	5.6
	SD	3.3	0.3	0.7	0.9	0.5
*P* value		*p* > .05	*p* < .001	*p* > .05	*p* < .001	*p* > .05

The last row shows the statistical significance of differences in the features between the single fascicular and double fascicular ATFL.

The abbreviations used in Table 4. ATFL, the anterior talofibular ligament; SD, standard deviation.

### The connection between the ATFL and CFL

**Figure 4:**
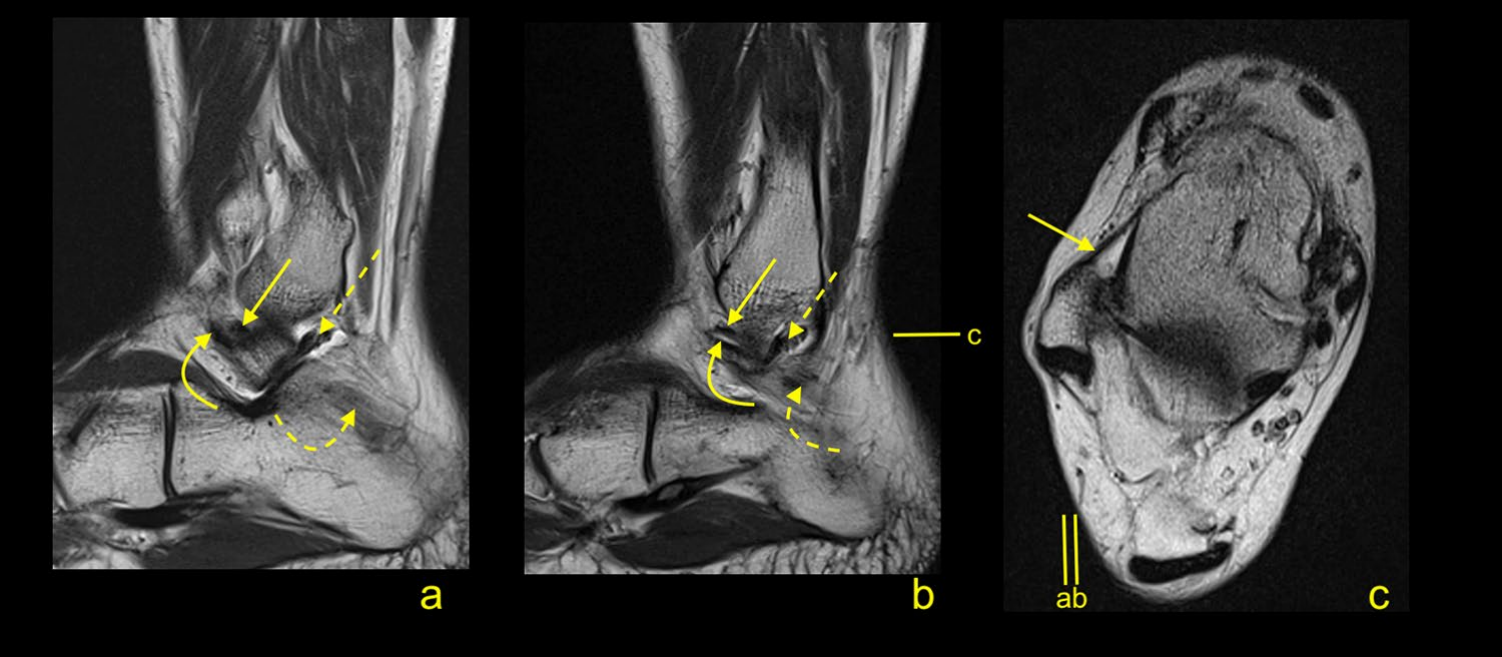
A 45-yeard-old patient with suspicion of a soft tissue tumor, which was not confirmed on the MRI. The double fascicular ATFL, two fascicles with the same diameter. Sagittal section (**a**,**b**) and transverse section (**c**). The S-ATFL (straight arrow), the I-ATFL (curved arrow), the PTFL (dashed arrow), and the CFL (curved dashed arrow). Levels of cross-sections are labeled on figures (**b**,**c**).

**Figure 5:**
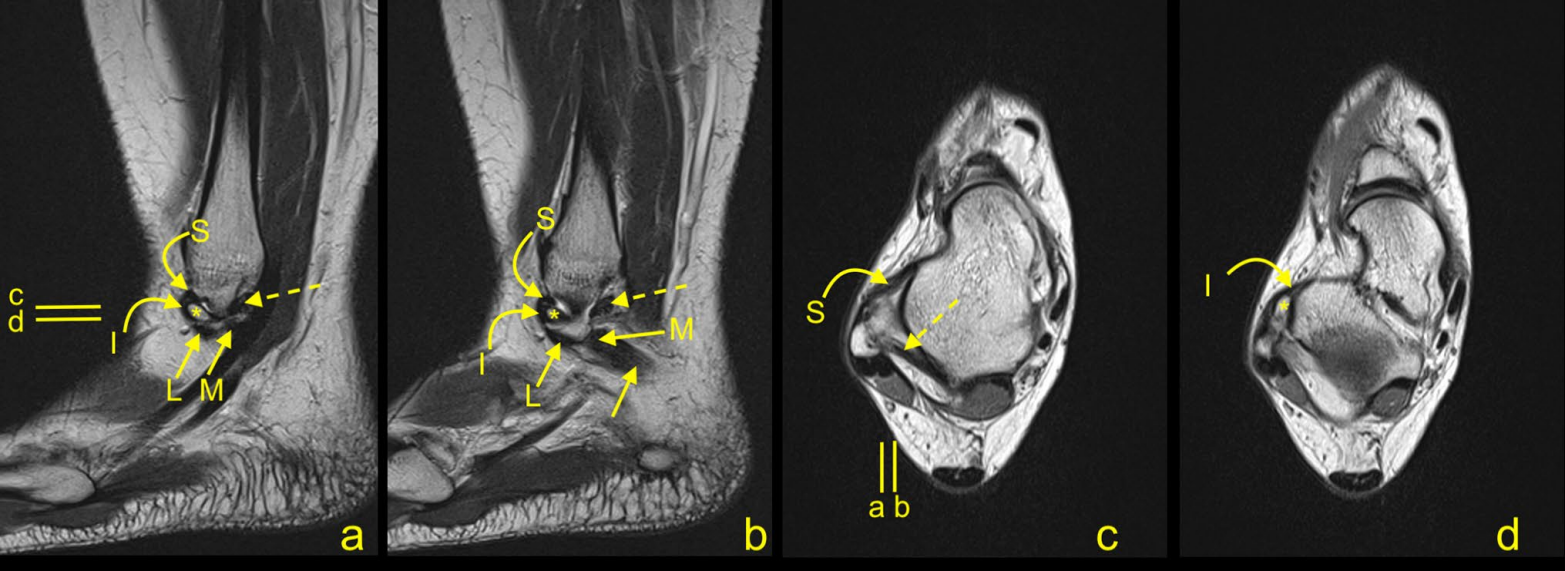
A 20-yeard-old patient with suspicion of ganglion originating from the talonavicular joint, which was not confirmed on the MRI. The presence of the os subfibulare does not change the anatomical relations of the ligaments. ATFL (curved arrow) and CFL (straight arrow). The M-CFL (M) runs anterior to communicate with the ATFL, while the L-CFL (L) attaches to the fibula.

The connection of the ATFL with the CFL was the most common one and was found in 148 cases (74.7%), Figs. 2, 3, 4, 5. The majority of the interconnections between the ATFL and CFL were observed in the groups of double fascicular ATFL (125 cases, 63.1%) and the double fascicular CFL (105 cases, 53%; Table 3). However, those differences were not statistically significant (Table 1). These interconnections were seen in the proximity to the fibular insertion of the ATFL and the CFL. This structure is often arciform and connecting the I-ATFL and the L-CFL (Figs. 2 and 3g).

### The connections of the ATFL and CFL with other structures

**Figure 6:**
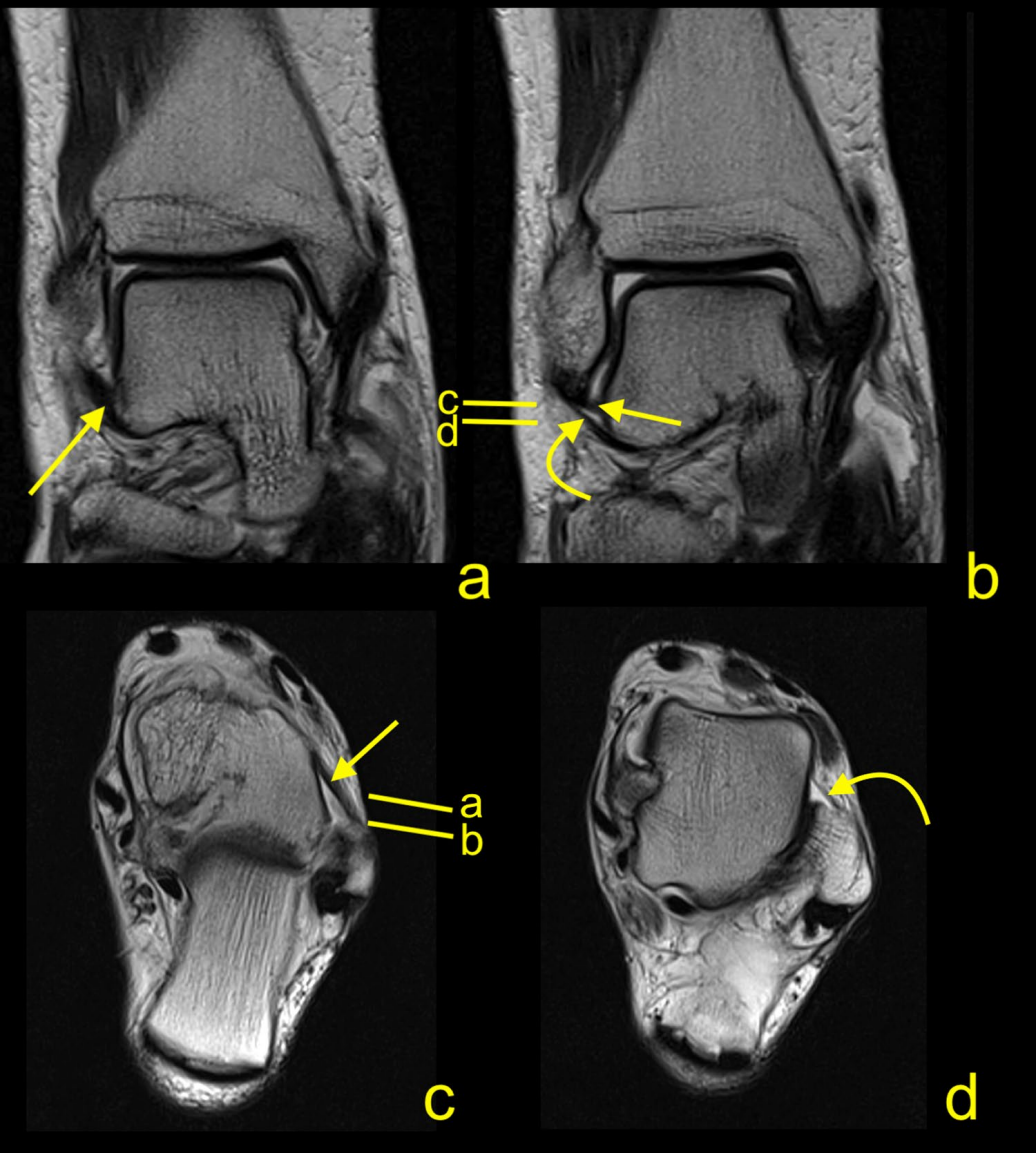
A 34-yeard-old patient with suspicion of the soft tissue tumor, which was not confirmed on the MRI. The double fascicular ATFL, two asymmetrical fascicles. Sagittal section (**a**,**b**) and transverse section (**c**). The S-ATFL (straight arrow), the I-ATFL (curved arrow), the PTFL (dashed arrow), and the CFL (curved dashed arrow). Levels of cross-sections are labeled on figures b and c.

The most connections with non-ligamentous structures were seen in groups of double fascicular ligaments (Figs. 4, 5, 6). In the group of the ATFL, the other most common connections were noticed with the SPR (26.8%), IER (24.2%), and PTS (14.6%), Table 3. Among the ATFL’s connections, only the connection with the PTS differed significantly between the single and double fascicular ligament (Table 3).

**Figure 7:**
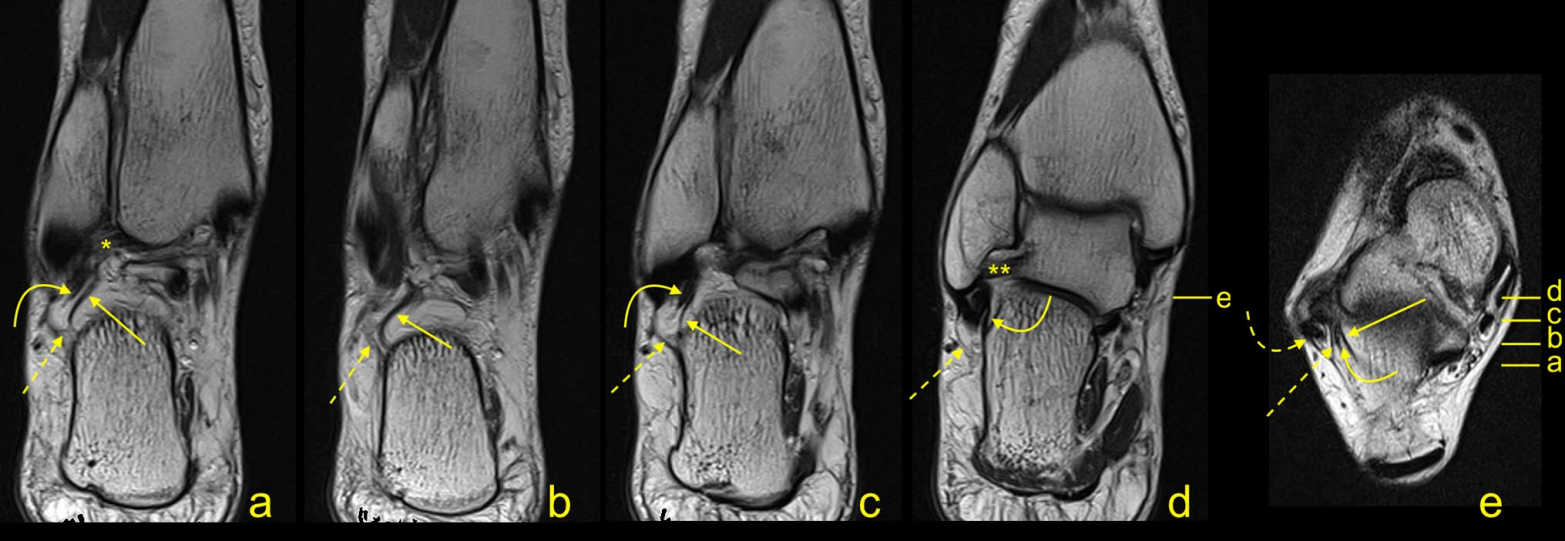
A 43-yeard-old patient with suspicion of a tumor. The MRI did not reveal a tumor in the soft tissue. The oblique sections (**a**–**d**) and the axial cross-section (**e**). Levels of cross-sections are labeled on figures (**d**,**e**). Connections of the CFL (curved arrow) with the FTCL (straight arrow), the PTS (dashed arrow), the superior peroneal retinaculum (dashed curved arrow), the PTFL (*), and the ATFL (**).

**Figure 8:**
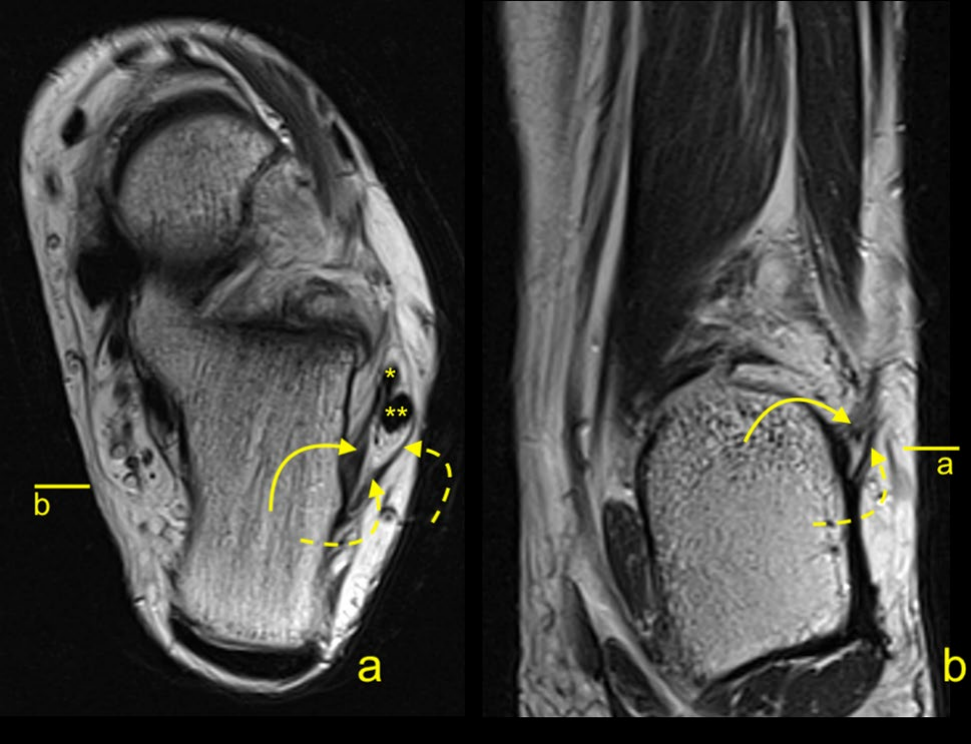
A 19-yeard-old patient with suspicion of the rheumatoid arthritis. No changes which correspond to this were found. The MRI did not reveal a tumor in the soft tissue. The communication between the CFL and the SPR. The axial section (**a**) and the corona section (**b**). Levels of cross-sections are labeled on both figures with lines and letters a and b. Connections of the CFL (curved arrow) with the superior peroneal retinaculum (dashed curved arrow), the peroneus brevis (*), and the peroneus longus (**).

In the group of the CFL, the other most common connections were with the PTFL (68.7%), the SPR (67.2%), and the FTCL (64.1%), Figs. 7 and 8. Among the CFL’s connections, only the connections with the PTFL, SPR, and PTS differed significantly between the single and double fascicular ligament (Table 3). We did not identify connections of the CFL with the AITFL and IER.

### The incidence of occurrence double fascicular ATFL and CFL

The presence of the double fascicular ATFL was identified in 148 cases (74.7%), of which in 101 cases (76.3%), two fascicles were completely separated while in 47 cases (23.7%) were incompletely separated. The single fascicular ATFL was identified in 50 cases (25.3%). The double fascicular CFL was recognized in 131 cases (66.2%), whereas a single fascicular CFL was seen in 67 cases (33.8%). In 84 cases (42.4%), the LTCL was present and branched from the CFL in 69 cases (34.8%), while in 15 cases (7.6%), it originated from the calcaneus as a separate structure.

**Figure 9:**
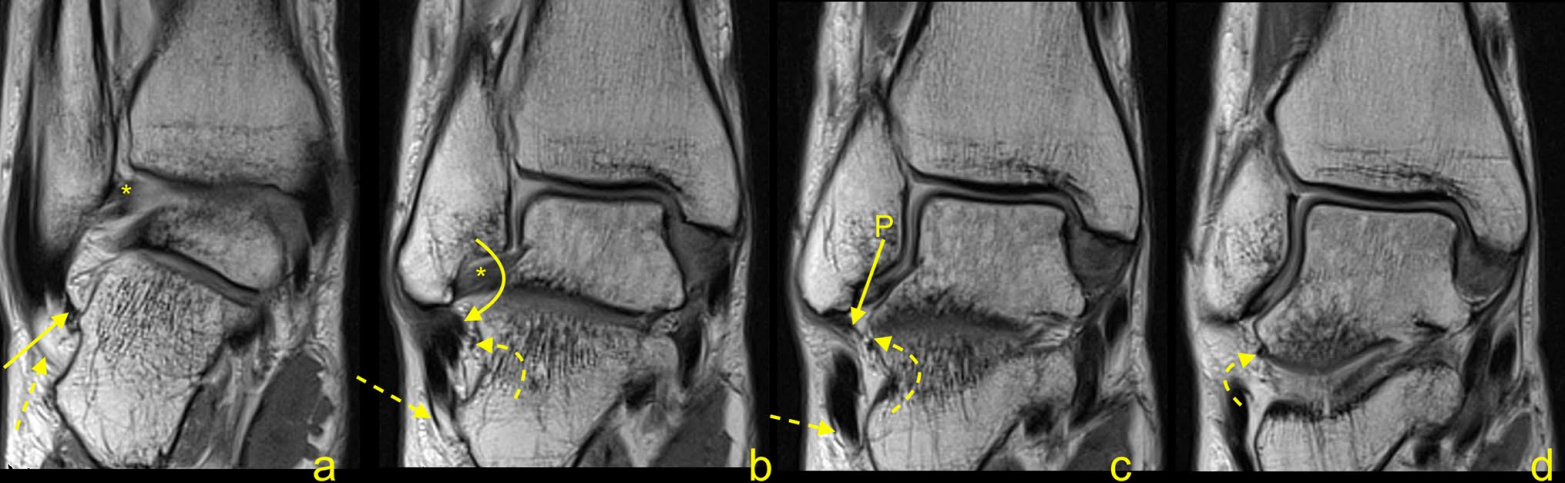
A 41-yeard-old patient with suspicion of a stress fracture of the distal tibia. No fractures were revealed on the MRI. The variably occurring accessory fascicle of the CFL inserts on the talus (curved dashed arrow). The CFL (straight arrow) in the distal part is not divided, and the proximal part (P) attaches to the lateral malleolus. The PTS (dashed arrow) and the PTFL (*).

The possibility to distinguish the medial (M-CFL) and the lateral (L-CFL) CFL fascicle was from the midportion and proximal to that. The L-CFL was always thicker than the M-CFL and had insertion on the inferoanterior outline of the apex of the lateral malleolus. We identified two variations of attachment of the M-CFL. The most often, one was seen on the fibula medial of the L-CFL in 84 cases (64.1%). The other one was found on the talus in 47 cases (23.7%) of which united with LTCL before it reaches the talus in 28 cases (14.1%) or ran directly to the talus in 19 cases (9.6%) (Fig. 9).

We revealed a statistically significant negative correlation between the CFL angle and TCA *r*(198) = − 0.25, *p* < 0.001. The differences in TCA were statistically significant, Table 5. In 52 cases (26.3%), the CFL angle was lower than 119 grades, which is the recommended cut-off for hindfoot^19^.

**Table 5:** The morphometry of the CFL, its bone attachments in the total number of CFL, and groups of single and double fascicular CFL, the CFL, and TCA angles.

		Length (mm)	Thickness of the midportion (mm)	The craniocaudal diameter of the midportion (mm)	Length of the fibular insertion (mm)	Length of the calcaneal insertion (mm)	The CFL angle (grades)	TCA (grades)
Totally CFL	Average	32.6	2.0	6.5	5.2	7.4	117.2	10.5
	Minimum	24.5	1.3	3.8	2.7	4.9	83	1.9
	Maximum	42.7	2.5	10.1	7.1	10.1	140	23
	SD	4.0	0.2	1.4	1.0	1.4	11.4	4.4
Single fascicular CFL	Average	32.82	1.97	6.29	5.24	7.03	118.16	13.1
	Min	28.10	1.60	3.80	3.10	4.90	98.00	9.8
	Max	42.70	2.10	9.20	6.40	8.90	133.00	19.4
	SD	4.73	0.17	1.61	0.93	1.26	10.45	3.6
Double fascicular CFL	Average	32.52	2.07	6.57	5.17	7.61	116.71	10.1
	Min	24.50	1.30	4.10	2.70	5.10	83.00	4.1
	Max	40.10	2.50	10.10	7.10	10.10	140.00	2.8
	SD	3.67	0.25	1.32	1.10	1.39	11.91	17.5
*P* value		*p* > .05	*p* < .001	*p* > .05	*p* > .05	*p* < .05	*p* > .05	*p* < .01

The last row shows the statistical significance of differences in the features between the single fascicular and double fascicular ATFL.

The abbreviations used in Table 5. CFL, the calcaneofibular ligament; SD, standard deviation; TCA, the tibiocalcaneal angle.

The differences in diameters between groups of single and double fascicular ATFL and CFL.

The average length of the ATFL was 24.2 ± 3.4 mm, the thickness in the midportion was 2.1 ± 0.3 mm, while the corresponding values for the CFL were 32.6 ± 4 mm and 2 ± 0.2 mm (Tables 4 and 5). Statistically significant differences between the single and double fascicular ATFL and CFL were noticed in thickness (Table 4). The superior fascicle of the ATFL (S-ATFL) was bigger in 122 cases (82.4%) than the I-ATFL, while in 26 cases (17.6%), the S-ATFL and the I-ATFL were at the same diameter. We did not notice any case where the I-ATFL was bigger.

The average breadth of the midportion of the ATLF was 5.1 ± 0.7 mm, and the corresponding value for the CFL was 6.5 ± 1.4 mm; however, without statistically significant differences (Tables 4 and 5). The average CFL angle was 117.2 ± 11.4 grades, while the TCA was 11.0 ± 4.4 grades, which was statistically significant (Table 5).

Bony attachments of the two fascicules of the ATFL, the talus and the fibula, were the same. The average craniocaudal length of the fibular insertions of the ATFL was 5.8 ± 0.9 mm, and the CFL was 5.2 ± 1 mm (Tables 4 and 5). However, statistically significant differences were noticed only between groups of the ATFL (Table 4). The average craniocaudal diameters of the talar insertion of the ATFL was 4.3 ± 0.6 mm, and the respective diameter of the calcaneal insertion of the CFL was 7.4 ± 1.4 mm, which was statistically significant (Tables 4 and 5).

## Discussion

The first contribution of the present study is that the connections with the non-ligamentous structures occur more often in groups of double fascicular ATFL or CFL. The second is that there are differences in diameter between single and double fascicular ligaments.

The nomenclature of the internal structure of a ligament is not clear and somewhat ambiguous. After other authors^1,10,25^, we implemented the term fascicule; however, there are no papers systematizing the nomenclature similar to the nomenclature of tendons^26^.

The occurrence of interconnections between the ATFL, CFL, and PTFL causes the formation of an anatomical and functional lateral fibulotalocalcaneal ligament complex^11^. It is located in the space between the medial side of the distal part of the lateral malleolus and the lateral outline of the talus^10,11^. Moreover, the footprint of the CFL and the ATFL is more confluent on the anterior border of the lateral malleolus^18^, also integrating bony attachments. The arciform fibers between the I-ATFL and the CFL^1^ may play a mechanical role in stabilizing the ankle when tension shifts between these two ligaments. After the S-ATFL is torn, the tearing continues via the arciform connection to the CFL, and the patient may develop ankle instability^11^. The extensive surgical dissection inferior to the lateral malleolus may cause injury to the arciform communication if it is preserved during trauma. Therefore, that should be considered during the surgery.

The connections between the ligament complex and adjacent non-ligamentous structures were revealed in our study, but to our knowledge, not studied previously in-depth. Furthermore, we revealed a significant correlation between the double fascicular structure of the ATLF and CFL with a higher occurrence of connections between the ATFL and CFL with non-ligamentous structures. The higher occurrence of communication in a group of double fascicular ligaments^1^ may, therefore, be due to development^27^. Moreover, anatomical studies show a relationship between the shape of the medial articular surface of the lateral malleolus and the connection between ATFL and PTFL, which may also indicate a developmental context.

Previously proven connections between the lateral ligament complex of the ankle with ligaments in the Kager’s fat pat may indicate the existence of some functional ligamentous-fascial units^28^ where the ligament complex is one part of the entirety. The presence of interconnections between the lateral ankle ligaments explain the concomitant nature of lateral ankle ligament injuries, making them like the deltoid ligament or spring ligament complex; despite having parts, it is challenging to separate them from each other^10,29^.

The AITFL connects with the ATFL stabilizing the position of the talus. The continuity of communication between the ATFL and AITFL preserves the anterior protrusion of the talus, which may cause friction with the inferior fascicle of the AITFL in patients with posttraumatic anterolateral hyperlaxity of the injured ATFL^25^. We noticed approximately a ten times lower occurrence of connections of the AITFL with ATFL when compared to the previous study, which was conducted on cadavers^25^. The wide differences between the anatomical studies based on dissections and research based on MRI^30^ were also noticed before in other ankle ligaments, which may be related to the method.

The presence of the connections of the CFL and the ATFL with the SPR and PTS may explain the posttraumatic instability of the peroneal tendons. It may be important for developing surgical techniques. Failure to recognize SPR injury may cause instability of the peroneal tendons and result in its rupture^31^. The existence of interconnections that lead to dysfunction of one structure may affect the function of the other. This clinical observation also confirms the significance of the described connections and their relationship with the double fascicular ATFL or CFL.

The fascicular feature of the ATFL was shown in anatomical studies^1,11^ and one anatomical-radiological study by Delfaut et al.^32^. Despite the amount of research, significant discrepancies in the frequency of the double fascicular ATFL from 25%^18^ to 100%^9^ are noticed. It is difficult to clearly define where such large discrepancies in the literature come from. It may be because some of these studies were done on small materials (Tables 1 and 2). In our study, the incidence of a single ATFL fascicle was comparable to most anatomical studies on larger material^1,3,8,33^. MRI is an appropriate method to detect abnormality and the presence of the fascicular structure of the ATFL^32^. We did not identify the tri- or multifascicular ligaments^9,20,33^. The third fascicle, if it is present, is the smallest of the ATFL fascicles and was reported in no more than 2%-12%, while some studies do not confirm its presence at all^3,22^. We believe that the absence of this rare variation in our result may be due to the MRI protocol or was a consequence of overdissection, which can be noticed when some of the anatomical studies are reviewed. The high occurrence of the double-fascicular structure of ATFL and CFL, together with the variability of connections, requires an update of the anatomical classification like it was done in other structures in the locomotor system^34^.

The presence of connections revealed in our study is also important for radiologists during the assessment of an ankle using an MRI after injury and for orthopedists in planning the reconstruction of ruptured ligaments^35^. The double fascicular structure of ATFL can be a diagnostic pitfall in MRI because the thin layers of connective tissue separating the bundles of the ligament have a slightly higher signal, imitating ligament abnormality^32^. Therefore, awareness of its occurrence is important for radiologists. The magic-angle effect may increase the intraligamentous signal, which may be seen on short echo time (TE) MRI sequences when a collagen structure is oriented at 55 grades to the main magnetic field^36^. Our study revealed the fascicular structure of the ATFL also on long-TE MRI sequences, which negates the presence of this artifact. No absence of the ATFL was detected, which was reported previously in 5%^3,22^.

The LTCL is a variable structure located in direct relation to the CFL. Most of the cases found in our study branched directly from the CFL, while in others, authors reported that it was never found as a separate structure^11^. The close relation to the CFL indicates that the LTCL, if present, is part of the lateral ankle ligament complex.

A ligament's fascicular structure allows different fiber orientations, functional differentiation, and better alignment of a joint. Due to interconnections, single smaller ligaments connect to form the larger anatomical-functional units resembling, for example, a cuff rotator in the shoulder. Functionally, this possibly results in better biomechanical joint stabilization^1^. We believe that the presence of the connections described by us is also crucial in training and preventing sports injuries. Strengthening one structure by the presence of connections may improve the function of others. However, further research is needed to confirm our hypothesis. We believe that our results provide sufficient anatomical background for further functional studies.

Differences between the diameters of the ligaments between the single and double fascicular were noticed. To our knowledge, there is no anatomic or radiologic paper about the correlation of the length, the width, and the thickness of the ATFL and the CFL with its fascicular nature. The length of the ATFL reported previously seemed to be circa 10 mm shorter when compared to studies on smaller materials^2,3,6,9,20^, whereas when compared to other authors who used larger material, our results were similar^1,22^ (Tables 1 and 2). Differences are probably related to the number of cases and methods of measurement with or without bony attachments^9,37^. It was reported that the wider and longer ligament’s fascicles are more prone to the pathological process than thinner ones^38^. Therefore, the diameters may have a clinical significance.

The thickness of the CFL shown in our study was consistent with what was previously reported^16,17^. The ATFL, in clubfeet, was reported to be much wider than in the two normal feet, which indicates a developmental background^12,27^. The width of the midportion assessed in our study is slightly smaller than reported previously^9,22,33^.

The thickness of the ligaments is one of the most important measurements for the radiologists because the ligamentous thickening and thinning is an MRI criteria for tears^32^. We reveled that ligaments’ dimensions are related to their fascicular structure. We used 3 T MRI because it allows for good resolution and excellent tissue differentiation^39^. To our knowledge, there is no cut-off value of the normal variants of the ATFL. The average thickness of the midportion of the ATFL revealed by us corresponds to the values reported before^22,40,41^ thus, we recommend using 2 mm as a cut-off value of the normal ligament. It is difficult to find studies on all dimensions of the ATFL and CFL on the same material, which makes comparison difficult (Tables 1 and 2).

To our knowledge, there have been no previous large-scale MRI studies regarding measurements of bony insertions. MRI examinations were performed for clinical purposes, so the assessment of the two dimensions of the insertion was somewhat difficult. Therefore, we included the largest diameter of the attachment-based on the known shape of the attachment^9,18,21,42^. Most of the previous studies provide the value of the area of insertion, which makes it harder to compare. However, some reference can be made. There is a discrepancy in the size ratio of the attachments reported in the literature. Some authors report that size of the talar insertion of the ATFL is bigger than the fibular one^9^, while the other reported exactly the opposite^18^. It is difficult to solve this problem based on our measurements. However, the craniocaudal diameter revealed in our study was larger in the fibula, where the attachment is more elongated, while the talar attachment is more oval^1,3,18^.

An unrecognized hindfoot valgus may cause a chronic deformity and secondary osteoarthritis. Initially, the patient experiences only the pain associated with overuse, and an MRI is often performed. Early identification of a valgus malalignment on an MRI may be crucial for the patient’s outcome. The CFL angle and the tension of the CFL change during movements and deformation. The cut-off value for the suspicion for a valgus hindfoot, ≤ 119 grades^19^, may be used with great sensitivity and specificity. The increased TCA is associated with a reduced CFL angle and may indicate a hindfoot^19^. Taking into consideration the presence of the connections between ligaments and non-ligamentous structures, fluctuations in tension in CFL translates to other structures causing changes in their function.

We acknowledge several limitations in our present study. Only one dimension of the bony attachments was measured. We believe that the full assessment of the bony attachments area and more accurate measurements may be possible when a special protocol with ultra-thin layers or with 3D sequences can be used. However, we believe that the MRI assessment of the fibular attachment may be difficult due to the multiple connections described^1,3,18^. The measurement errors may be related to the cutting angle and the measurement point of the single slice image. The retrospective character may also be a limitation of the study.

The presence of the double fascicular structure of the ATFL and CFL is related, more often, with the occurrence of interconnections between these two ligaments as well as with the adjacent structures.

## Abbreviations

AITFL: The anterior inferior tibio-fibular ligament
ATFL: The anterior talofibular ligament
CFL: The calcaneofibular ligament
FTCL: The fibulotalocalcaneal ligament
I-ATFL: The interior fascicle of the ATFL
IER: The inferior extensor retinaculum
L-CFL: The lateral fascicle of the CFL
LTCL: The lateral talocalcaneal ligament
M-CFL: The medial fascicle of the CFL
PTFL: The posteriori talofibular ligament
PTS: The peroneal tendon sheath
S-ATFL: Superior fascicle of the ATFL
SD: Standard deviation
SPR: The superior peroneal retinaculum
TCA: The tibiocalcaneal angle
TE: Echo time
